# SARS-CoV-2 M Protein Facilitates Malignant Transformation of Breast Cancer Cells

**DOI:** 10.3389/fonc.2022.923467

**Published:** 2022-06-07

**Authors:** Hoai-Nga Thi Nguyen, Marie Kawahara, Cat-Khanh Vuong, Mizuho Fukushige, Toshiharu Yamashita, Osamu Ohneda

**Affiliations:** Graduate School of Comprehensive Human Science, Laboratory of Regenerative Medicine and Stem Cell Biology, University of Tsukuba, Tsukuba, Japan

**Keywords:** COVID-19, breast cancer, metastasis, EMT, SARS-CoV-2

## Abstract

Coronavirus disease 2019 (COVID-19) has spread faster due to the emergence of SARS-CoV-2 variants, which carry an increased risk of infecting patients with comorbidities, such as breast cancer. However, there are still few reports on the effects of SARS-CoV-2 infection on the progression of breast cancer, as well as the factors and mechanisms involved. In the present study, we investigated the impact of SARS-CoV-2 proteins on breast cancer cells (BCC). The results suggested that SARS-CoV-2 M protein induced the mobility, proliferation, stemness and *in vivo* metastasis of a triple-negative breast cancer (TNBC) cell line, MDA-MB-231, which are involved in the upregulation of NFκB and STAT3 pathways. In addition, compared to MDA-MB-231 cells, the hormone-dependent breast cancer cell line MCF-7 showed a less response to M protein, with the protein showing no effects of promoting proliferation, stemness, and *in vivo* metastasis. Of note, coculture with M protein-treated MDA-MB-231 cells significantly induced the migration, proliferation, and stemness of MCF-7 cells, which are involved in the upregulation of genes related to EMT and inflammatory cytokines. Therefore, SARS-CoV-2 infection might promote the ability of aggressive BCC to induce the malignant phenotypes of the other non-aggressive BCC. Taken together, these findings suggested an increased risk of poor outcomes in TNBC patients with a history of SARS-CoV-2 infection, which required a long-term follow-up. In addition, the inhibition of NFκB and STAT3 signaling pathways is considered as a promising candidate for the treatment of worsen clinical outcomes in TNBC patients with COVID-19.

## Introduction

Coronavirus disease 2019 (COVID-19) has spread globally since it was first detected in December 2019, resulting in a pandemic that has impacted most nations and resulted in about 250 million illnesses and 5 million deaths (1). COVID-19 is a severe infectious respiratory disease caused by SARS-CoV-2, a new highly contagious virus from the family coronaviridae, like SARS-CoV and MERS-CoV (2). After infection, SARS-CoV-2 causes acute powerful inflammation, which leads to a high mortality rate (2). Although urgent vaccination programs in many countries have helped reduce the number of new cases and the severity of symptom in such cases, the long-term impact of SARS-CoV-2 infection on human health still needs to be investigated.

Among COVID-19 patients, COVID-19–infected cancer patients appear to be more vulnerable than others. Cancer patients have a higher risk of infection, more severe symptoms, and higher mortality rate than non-cancer patients (3). Previous reports have shown that cancer patients had a high incidental diagnosis of SAR2-CoV-2 infection and also suffered worse outcomes than the general population (4–10). Defects in their immune system due to chemotherapy and radiotherapy not only facilitate infection with SARS-CoV-2 (3) but also lead to difficulty enjoying the protective effects of COVID-19 vaccination (11). Furthermore, cancer-related chronic inflammation contributes to more severe symptoms when patients do get infected (3).

In cancer and COVID-19, inflammation is a common characteristic of the pathophysiology. Cytokine storm is a systemic hyperactive immune status characterized by massive cytokine release and is seen in both SARS-CoV-2 infection and cancer (12). Among the cytokines involved in cytokine storm, interleukin-6 (IL6) and tumor necrosis factor α (TNFα) are key proinflammatory cytokines, playing major driver roles in the acute immune response during severe COVID-19 infection (12). IL6 stimulates cancer cell activities and features, such as proliferation, mesenchymal transformation, metastasis, stemness and immune evasion (13, 14). Furthermore, SARS-CoV-2 infection-related cytokine storm and systemic inflammation can induce oxidative stress, DNA damage and genetic instability in normal cells, which trigger the development of benign tumors and malignant transformation in the presence of oncoviruses (15). In addition, Francescangeli et al. suggested that the prolonged inflammation, leukocyte hyperactivation, T-cell impairment and thrombocytosis associated with COVID-19 may create a suitable microenvironment to reawaken dormant cancer cells, particular those with stem-like characteristics that survive after chemotherapy or radiotherapy and have the potential to cause recurrence or metastasis (13, 14). Moreover, Wei et al. also reported a significant increase in serum cancer biomarkers levels in critical cases of COVID-19 disease (16), implying a correlation between SARS-CoV-2 infection and tumorigenesis. Therefore, studying the effects of COVID-19 on cancer is an urgent need in the field of preventive healthcare of cancer patients.

Breast cancer is one of the most frequent cancer types among SARS-CoV-2-infected cancer patients (6, 17, 18). Breast cancer patients infected with SARS-CoV-2 might develop new metastases, progression and death due to tumor progression (8, 9). Therefore, in the present study, we aimed to examine the correlation between SARS-CoV-2 infection and breast cancer progression by studying the effects of SARS-CoV-2 proteins on the phenotypes of different types of human breast cancer cells (BCC), including aggressive MDA-MB-231 cells and non-aggressive MCF-7 cells. In addition, the impact of SARS-CoV-2 proteins on the paracrine effects of aggressive BCC on non-aggressive BCC were also examined.

## Materials and Methods

### Breast Cancer Cell Culture and Induction With SARS-CoV-2 Protein

The human breast cancer cell lines MDA-MB-231 (ATCC HTB-26) and MCF-7 (ATCC HTB-22) were cultured in Iscove’s modified Dulbecco’s (IMDM) medium (Gibco, Waltham, MA, USA) supplemented with 5% fetal bovine serum (FBS) (Sigma-Aldrich, St. Louis, MO, USA) and 1% penicillin/streptomycin (Thermo Fisher Scientific, Waltham, MA, USA). Cells were maintained in humidified incubator with 5% CO_2_ at 37°C. The medium was changed every two days. On reaching 80% confluence, the cells were trypsinized and subcultured at an initial density of 3.8×10^4^cells/ml in culture medium.

A total of 5×10^5^ cells/ml were then treated by SARS-CoV-2 peptivator Peptide Pools Prot_M (M protein; PepTivator SARS-CoV-2 Prot M, 130-126-702, Miltenyi Biotec, Cologne, Germany), SARS-CoV-2 peptivator Peptide Pools Prot_S (S protein; PepTivator SARS-CoV-2 Prot S, 130-126-700, Miltenyi Biotec) and SARS-CoV-2 peptivator Peptide Pools Prot_N (N protein; PepTivator SARS-CoV-2 Prot N, 130-126-698, Miltenyi Biotec) at concentrations of 60 pmol/ml for 24 h before further analyses.

### The Migration Assay

MDA-MB-231 and MCF-7 cells were seeded into each well of a 24-well plate at 2×10^5^ cells/400 µl/well and 1.5×10^5^ cells/300 µl/well, respectively, in cultured medium and incubated for 1 hour before being treated with M protein, S protein and N protein. After 24 h of treatment, Mitomycin C solution (Nacalai Tesque, Kyoto, Japan) was added to the medium at 10 μg/ml for 2 h. A single scratch wound was then created using a 100-µl micropipette tip. The cultured medium was removed and changed to IMDM 0.25% FBS. Pictures were taken right after scratching and 24 h after scratching using a Keyence BZ-X710 microscope system (Keyence Corporation, Osaka, Japan). The migration distance (μm) at 0 and 24 h after wounding was measured using the ImageJ software program (NIH, Bethesda, MD, USA).

### The Matrigel Invasion Assay

A suspension of MDA-MB-231 or MCF-7 cells (1×10^5^ cells/200 μl) in IMDM medium was seeded onto a BD Matrigel Basement Membrane Matrix (BD Biosciences, Franklin Lakes, NJ, USA)‐coated 8‐μm BD Falcon cell culture insert transwell (BD Biosciences). A total of 400 μl of IMDM supplemented with 10% FBS was added to the lower compartments of each chamber. The cells were treated with M protein for 24 h. After removing the cells remaining inside the transwell with a cotton swab, the bottom surface of each transwell membrane were then fixed in 4% paraformaldehyde in two minutes, permeabilized in methanol in 20 minutes. Then, the fixed transwell membranes were dipped in Hematoxylin in 30 seconds and washed in distilled water two times, following by dipping in Eosin in 20 seconds and washing in distilled water two times. The membranes were observed under microscope immediately after staining. Five random pictures were taken for each transwell, and the average number of cells was counted using the ImageJ software program. The invasion rate was calculated as follows:


$$\begin{array}{l} \text{Invasion percentage = Average number of cells in coated transwell}\times 100/\text{Average}\\ \text{number of cells in uncoated transwell} \end{array}$$

### The Mammosphere Formation Assay

A suspension of MDA-MB-231 or MCF-7 cells (9.5×10^3^ cells/2 ml) was mixed in MammoCult Basal medium (StemCell Technologies Inc., Vancouver, Canada) containing heparin and hydrocortisone and cultured for 5 days in a 6-well cell culture plate with an ultra-low-attachment surface (Corning; Corning, NY, USA). The mammosphere (diameter ≥100 μm)-forming efficiency (MSFE) was calculated as follows:


MSFE (%) = number of mammospheres × 100 / number of seeded cells

### The Coculture Assay

MCF-7 cells were seeded into each well of a 24-well plate at 1×10^5^ cells/200 µl/well in culture medium and incubated for 2 hours before coculture. A suspension of MDA-MB-231 or MCF-7 cells (1×10^5^ cells/200 μl) seeded onto a 3‐μm BD Falcon cell culture insert transwell (BD Biosciences) was then inserted into an MCF-7 cell-seeded 24-well plate. The cells were treated with M protein for 48 h before undergoing further examinations.

### Collection of Conditioned Medium

MDA-MB-231 cells or MCF-7 at the density of 5×10^5^ cells/ml were treated by M protein at concentrations of 60 pmol/ml for 24 h, then the conditioned medium was collected. The conditioned medium was centrifuged at 1000 rpm in 5 minutes, following by another step at 2000 rpm in 20 minutes to remove the cell death and cell debris. In order to inhibit the inflammatory cytokines, neutralizing antibodies were added to conditioned medium, including human anti-TNFα antibody (MAB210, R&D Systems, Minneapolis, MN, USA), anti-IL6 antibody (MAB2061, R&D Systems) and anti-IL8 antibody (MAB208, R&D Systems) at concentrations of 10 μg/ml, 5 μg/ml and 5 μg/ml respectively.

### The Proliferation Assay

MDA-MB-231 and MCF-7 cells were seeded into each well of a 96-well plate at 1×10^4^ cells/100 µl/well in culture medium and incubated for 1 hour before being treated with M protein. After 24, 48 and 72 h of treatment, the cell density was determined using a Cell Counting Kit (Dojindo Molecular Technologies, Kumamoto, Japan) 1 h before the absorbance was measured at a wavelength of 450 nm (OD_450nm_).

### Quantitative Reverse Transcription (qRT) PCR Gene Expression Analyses

To investigate the gene expression at the transcriptional level, qRT-PCR was carried out. MDA-MB-231 and MCF-7 cells were seeded into each well of a 6-well plate at 5×10^5^ cells/1 ml/well in culture medium and incubated for 1 hour before being treated with M protein. After 24 hours, total RNA was isolated using Sepasol-RNA Super G (Nacalai Tesque) according to the instruction of the manufacturer. 2 μg of total RNA sample was reverse transcribed into cDNA using RT-PCR ReverTra Ace qPCR RT kit (Toyobo, Kita, Osaka, Japan). 2 µl of cDNA templates was subjected to realtime PCR amplification using THUNDERBIRD SYBR qPCR Mix (Toyobo) in Real-time PCR system QuantStudio 5 (Thermo Fisher Scientific). The qPCR program comprised an initial denaturation step at 95°C for 10 minutes, followed by 40 cycles of denaturation step at 95°C for 15s, annealing and extension step at 60°C for 30s. The sequences of qPCR primers for analysis were listed in **Table 1**. The expression levels of target genes were analyzed using the ΔΔCt method and normalized to the expression level of internal control housekeeping gene *ACTB* (β-actin) in each sample by the formula 2^-ΔCt^.

**Table 1:** Primer sequences.

Primer	Sequence
β-Actin	CTCGCCTTTGCCGATCC
	TCTCCATGTCGTCCCAGTTG
Vimentin	CCGTTGAAGCTGCTAACTACCAAGAC
	GTGGGTATCAACCAGAGGGAGTGAAT
N-Cadherin	GTGGAGGAGAAGAAGACCAGGACTATG
	CTAACAGGGAGTCATATGGTGGAGCTG
IL6	ACAAGAGTAACATGTGTGAAAGCAG
	TATACCTCAAACTCCAAAAGACCAG
IL8	GAGAGTGATTGAGAGTGGACCAC
	CACAACCCTCTGCACCCAGTTT
TNFα	TCCTTCAGACACCCTCAACC
	AGGCCCCAGTTTGAATTCTT
Snail	AACTACAGCGAGCTGCAGGACTCTAA
	CCTTTCCCACTGTCCTCATCTGACA
Twist	AGCCGCAGAGACCTAAACAA
	CACGCCCTGTTTCTTTGAAT
Slug	CTCCTCTTTCCGGATACTCCTCATCT
	CCAGGCTCACATATTCCTTGTCACAG
Zeb1	CAGCTCTGGGTGGAGAAGAC
	CCTGACCCACTTCCAACAGT
HIF-1α	TTACCGAATTGATGGGATATGAG
	TCATGATGAGTTTTGGTCAGATG
ACE2	AGGAGGTCTGAACATCATCAGTG
	GGGATCAGAGATCGGAAGAAGAAA
TMPRSS2	AATCGGTGTGTTCGCCTCTAC
	CGTAGTTCTCGTTCCAGTCGT

### Western Blotting

MDA-MB-231 and MCF-7 cells were seeded into 10cm dish at 3.2×10^6^ cells/6.4 ml/dish in culture medium and incubated for 1 h before being treated with M protein. After 24 h, cells were harvested, and nuclear proteins were extracted using RIPA buffer (25 mM Tris, 150 mM NaCl, 1% NP-40, 1% sodium deoxycholic acid, 0.1% SDS) for 30 min; samples were then centrifuged at 15,000 rpm at 4°C for 10 min. The protein concentration was measured using the Bradford method (Biorad, Hercules, CA, USA).

Protein samples were denatured at 95°C for 3 minutes in sodium dodecyl sulfate (SDS) loading buffer (Wako Pure Chemical, Osaka, Japan) and subjected to SDS-polyacrylamide gel electrophoresis (PAGE) (50 μg per sample) and electrotransferred to PVDF membranes (Merck Millipore, Burlington, MA, USA). Membranes were then incubated with primary antibodies, including rabbit anti-pan-STAT3 (8204S; Cell Signaling Technology, Danvers, MA, USA) and rabbit anti-phosphorylated STAT3 (pSTAT3) (8204S; Cell Signaling Technology) at 1:1000 dilution. Horseradish peroxidase (HRP)-conjugated goat anti-rabbit IgG (Thermo Fischer Scientific) was used as a secondary antibody at 1:10000 dilution. Signals were detected by chemiluminescence HRP substrate (Merck Millipore) in an Image Quant LAS 4000 system (GE Healthcare, Chicago, IL, USA) and analyzed using the ImageJ software program.

### The *In Vivo* Metastasis Assay

Female C57BL/6J mice were bred under specific-pathogen-free (SPF) conditions. All experimental procedures were approved by the University of Tsukuba Institute Animal Care and Use Committee. MDA-MB-231 and MCF-7 cells were seeded into each well of a 6-well plate at 5×10^5^ cells/1 ml/well in culture medium and incubated for 1 hour before being treated with M protein. After 24 h, cells were harvested and resuspended in phosphate-buffered saline (PBS) before injection. The cell suspension (2 ×10^5^ cells/300 μl) was injected into the tail vein, and mice were injected with Cyclosporin‐A (Sigma‐Aldrich) every day for the first week and every 2 days for the second week (200 μl per mouse) for immunosuppression.

After 14 days, the mice were sacrificed by cervical dislocation, and the lungs were collected, fixed with 4% paraformaldehyde (Wako Pure Chemical), and turned into frozen sections. The lung sections were stained by Hematoxylin–Eosin. All sections of each sample were observed under a microscope to find all tumors foci. The tumor foci area was measured by the ImageJ software program.

### Statistical Analyses

The results were described as the mean ± standard deviation (SD). Differences were analyzed using the Mann Whitney U-test of the GraphPad Prism 5 software program (GraphPad Software Inc., San Diego, CA, USA). Differences were considered to be significant if P value of ≤0.05.

## Results

### Different Responses of TNBC Cells and Hormone Dependent Cells to SARS-CoV-2 M Protein

**Figure 1:**
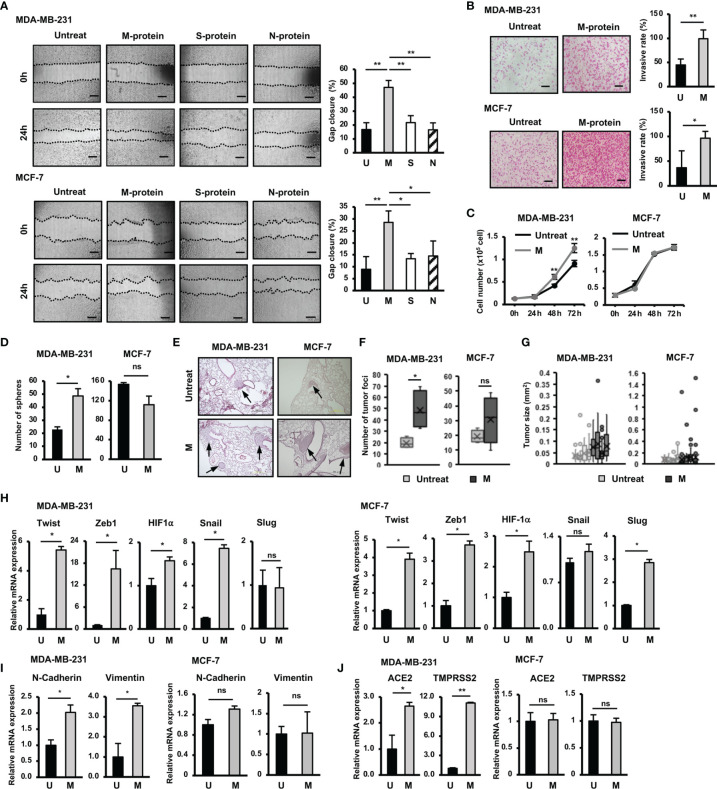
Responses of BCC to SARS-CoV-2 protein. **(A)** The migration of BCC in response to SARS-CoV-2 proteins. **(B)** The BCC invasion assay in response to M protein. **(C)** The proliferation of BCC in response to M protein. **(D)** The mammosphere assay of BCC in response to M protein. **(E)** Lung sections from an *in vivo* metastasis assay of BCC, arrows indicate tumors in lungs. **(F)** Number of tumor foci from an *in vivo* metastasis assay. **(G)** Tumor sizes from an *in vivo* metastasis assay in individual mice; each dot indicates a tumor. **(H)** The mRNA expression of genes related to EMT process in BCC. **(I)** The mRNA expression of EMT markers in BCC. **(J)** The mRNA expression of SARS-CoV-2 binding receptor genes in BCC. U, untreated BCC; M, M protein-treated BCC. The scale bars indicate 500 µm. Each value represents the mean ± SD of triplicate experiments. (ns, no significance; p > 0.05; *p ≤ 0.05; **p ≤ 0.01).

Firstly we examined the effects of SARS-CoV-2 proteins, including membrane protein (M protein), spike protein (S protein) and nucleocapsid protein (N protein) on the migratory ability of aggressive breast cancer cells (BCC), MDA-MB-231 cells, and non-aggressive BCC, MCF-7 cells. The results showed that compared with S and N proteins, M protein shows a significantly greater ability to induce the migration of both MDA-MB-231 and MCF-7 cells (MDA-MB-231: 2.8-fold increase, MCF-7: 1.6-fold increase, **Figure 1A**). Therefore, we next examined the effects of M protein on the other phenotypes of BCC, including the invasion, proliferation, and stemness. As a result, M protein induced the invasion through Matrigel in both MDA-MB-231 and MCF-7 cells (MDA-MB-231 cells: 2.25-fold increase MCF-7 cells: 2.6-fold increase, **Figure 1B**). However, while M protein induced proliferation and sphere formation in MDA-MB-231 cells (proliferation: 1.4-fold increase after 72 h of treatment, sphere formation: 2.1-fold increase, **Figures 1D, E**), MCF-7 cells showed no marked induction of proliferation or stem-like sphere formation after treatment with M protein (**Figures 1C, D**).

We next examined the effects of M protein on the *in vivo* metastasis of BCC in a lung metastatic mouse model. BCC (including those treated by M protein) were intravenously injected into mice *via* the tail vein, and then the number of tumor foci and the size of tumors in the lungs were examined (**Figure 1E**). As a result, mice injected with M protein-treated MDA-MB-231 cells showed significantly more tumor foci in the lungs (2.5-fold increased, **Figure 1F**) than those injected with untreated MDA-MB-231 cells. In addition, M protein-treated MDA-MB-231 cells tended to form larger tumors with a greater tumor size variability in mouse lungs than untreated MDA-MB-231 cells (**Figure 1G**). In contrast to MDA-MB-231 cells, MCF-7 cells showed no induced metastatic ability *in vivo* after treatment with M protein. Mice injected with M protein-treated MCF-7 cells showed no significant difference in the number of tumor foci (**Figures 1E, F**) from those injected with untreated MCF-7 cells, and although there were some remarkably large tumors (>0.4 mm^2^) in mice injected with M protein-treated MCF-7 cells, the median tumor size did not remarkably increase compared to those injected with untreated MCF-7 cells (**Figure 1G**).

Epithelial to mesenchymal transition (EMT) plays an important role in tumor progression and metastasis (19). Upon EMT, carcinoma cells lose epithelial marker expression and cell polarity and instead acquire the mesenchymal morphology, mobility and invasion capabilities critical for tumor invasion and metastasis (19). In addition, cancer cells obtain stem cell-like characteristics through EMT, which facilitates cancer relapse and metastasis (20). Therefore, we next examined the effects of M protein on the expression of genes related to EMT, proliferation and stemness of BCC. The results showed that M protein upregulated the expression of genes related to stemness and EMT, such as Twist (5.4-fold increase), Zeb1 (16-fold increase), HIF-1α (1.5-fold increase) and Snail (7.2-fold increase) in MDA-MB-231 cells, and Twist (3.9-fold increase), Zeb1 (3.7-fold increase), HIF-1α (2.5-fold increase) and Slug (2.7-fold increase) in MCF-7 cells (**Figure 1H**). In addition, while MDA-MB-231 cells treated with M protein showed an increased expression of mesenchymal markers, including N-Cadherin (2-fold increase) and Vimentin (3.5-fold increase) (19), MCF-7 cells showed no altered expression of these genes by M protein (**Figure 1I**). Interestingly, the expressions of ACE2 and TMPRSS2, the binding receptors of SARS-CoV-2, were upregulated by the induction of M protein in MDA-MB-231 cells (ACE2: 2.8-fold increase, TMPRSS2: 11.3-fold increase) but not in MCF-7 cells (**Figure 1J**).

Taken together, these data suggested different responses to SARS-CoV-2 M protein between TNBC cells and hormone-dependent BCC. While M protein induced the *in vitro* migration and invasion of both MDA-MB-231 and MCF-7 cells, only MDA-MB-231 cells showed the promotion of proliferation, stemness and *in vivo* metastasis in response to M protein.

### Involvement of the NFκB Pathway in the Induction of the Upregulation of EMT Genes and Migration of BCC by SARS-CoV-2 M Protein

**Figure 2:**
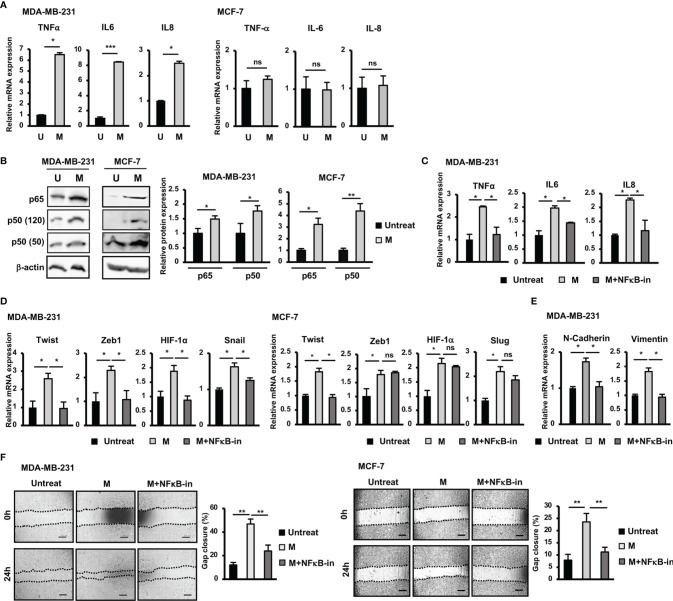
NFκB pathway was involved in the upregulation of EMT genes and the induction of migration of BCC by SARS-CoV-2 M protein. **(A)** Gene expression of inflammatory cytokines in BCC treated with M protein. **(B)** Protein expression of p65 and p50 in BCC treated with M protein. **(C)** The mRNA expression of inflammatory cytokines in BCC in the presence of M protein and NFκB inhibitor. **(D)** The mRNA expression of genes related to EMT process in BCC in the presence of M protein and NFκB inhibitor. **(E)** The mRNA expression of EMT markers in MDA-MB-231 cells treated with M protein in the presence of NFκB inhibitor. **(F)** Migration of BCC treated with M protein in the presence of NFκB inhibitor. BAY11-7082 10 μM was used as NFκB inhibitor. U, untreated BCC; M, M protein-induced BCC. The scale bars indicate 500 µm. Each value represents the mean ± SD of triplicate experiments. (ns, no significance; p > 0.05; *p ≤ 0.05; **p ≤ 0.01; ***p ≤ 0.001).

SARS-CoV-2 infection is associated with cytokine storm characterized by the massive release of inflammatory cytokines, including IL6, TNFα and IL8 (12, 21). In addition, previous studies have suggested a relationship between inflammatory cytokines and EMT in cancer cells (22). Therefore, we next examined the expression of inflammatory cytokines treated with M protein in BCC. As shown in **Figure 2A**, after treatment with M protein, MDA-MB-231 cells showed the significant upregulation of TNFα, IL6 and IL8 (TNFα: 6.6-fold increase, IL6: 9.4-fold increase; IL8: 2.6-fold increase). However, M protein showed no effect of inducing the expression of these inflammatory cytokines in MCF-7 cells (**Figure 2A**).

Numerous studies have reported the role of the NFκB signaling pathway in the upregulation of inflammatory cytokines, such as IL6, IL8, and TNFα (23). In addition, NFκB activation in breast cancers results in EMT and a stem-like phenotype (23). Therefore, we next examined the involvement of NFκB signaling pathway in the upregulation of inflammatory and EMT genes in M protein-treated BCC. As shown in **Figure 2B**, both MDA-MB-231 and MCF-7 cells showed an increase in p50 (MDA-MB-231: 1.8-fold increase, MCF-7: 3.2-fold increase) and p65 (MDA-MB-231: 1.5-fold increase, MCF-7: 4.4-fold increase) in response to M protein. Of note, in the presence of an NFκB inhibitor, M protein-treated MDA-MB-231 cells showed the downregulation of inflammatory cytokines (**Figure 2C**).

Next, we examined the effect of treatment with an NFκB inhibitor on the expression of EMT genes which were upregulated by M protein in MDA-MB-231 and MCF-7 cells. As shown in **Figure 2D**, treatment with an NFκB inhibitor impaired the expression of EMT genes upregulated by M protein in MDA-MB-231, such as Twist (2.2-fold decrease), Zeb1 (2.5-fold decrease), HIF-1α (2-fold decrease) and Snail (1.4-fold decrease). However, while treatment with an NFκB inhibitor induced the downregulation of Twist expression (2-fold decrease) in M protein-treated MCF-7 cells, no effects on the other genes, such as Zeb1, Slug or HIF-1α, were observed (**Figure 2D**). In addition, treatment with an NFκB inhibitor resulted in the downregulation of mesenchymal markers, including N-Cadherin (1.7-fold decrease) and Vimentin (1.8-fold decrease) in M protein-treated MDA-MB-231 (**Figure 2E**). We next examined the effects of NFκB inhibitor on the migration of M protein-treated BCC. As a result, treatment with NFκB inhibitor significantly decreased the migratory ability of both M protein-treated MDA-MB-231 cells and M protein-treated MCF-7 cells (MDA-MB-231: 2-fold decrease, MCF-7: 2.1-fold decrease, **Figure 2F**).

Taken together, these results suggested that M protein significantly induced the expression of inflammatory cytokines in MDA-MB-231 cells but not MCF-7 cells, which was involved in the upregulation of the NFκB pathway. Of note, the NFκB pathway was also highly involved in the M protein-induced migration of both MDA-MB-231 and MCF-7 cells.

### Contribution of STAT3 Pathway Activation to the Induction of the Upregulation of EMT Genes and Migration of BCC by SARS-CoV-2 M Protein

**Figure 3:**
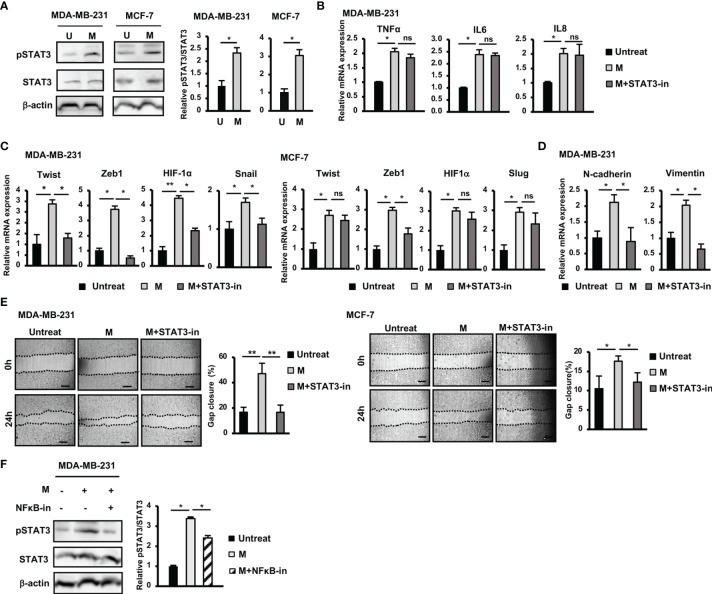
Activation of STAT3 pathway contributed to the upregulation of EMT genes and the induction of migration of BCC by SARS-CoV-2 M protein. **(A)** The phosphorylation of STAT3 in BCC treated with M protein. **(B)** Gene expression of inflammatory cytokines in MDA-MB-231 cells treated with M protein in the presence of STAT3 inhibitor. **(C)** EMT-related gene expression in BCC treated with M protein in the presence of STAT3 inhibitor. **(D)** The mRNA expression of EMT markers in MDA-MB-231 cells treated with M protein in the presence of STAT3 inhibitor. **(E)** Migration of BCC treated with M protein in the presence of STAT3 inhibitor. **(F)** The phosphorylation of STAT3 in MDA-MB-231 in the presence of M protein and the NFκB inhibitor. Galiellalactone 100 ng/ml (SC-202165; Santa Cruz Biotechnology) was used as a STAT3 inhibitor. BAY11-7082 10 μM was used as an NFκB inhibitor. U, untreated BCC; M, M protein-induced BCC. The scale bars indicate 500 µm. Each value represents the mean ± SD of triplicate experiments. (ns, no significance; p > 0.05; *p ≤ 0.05; **p ≤ 0.01).

In addition to the NFκB pathway, the Jak/STAT3 pathway is also reportedly involved in the upregulation of genes related to EMT in BCC (24). Therefore, we examined the role of the STAT3 pathway in the responses of BCC to M protein. The results showed that treatment with M protein significantly activated the phosphorylation of STAT3 in both MDA-MB-231 and MCF-7 cells (MDA-MB-231: 2.3-fold increase, MCF-7: 3-fold increase, **Figure 3A**).

In addition, while treatment with a STAT3 inhibitor showed no marked effects on the expression of inflammatory cytokines in MDA-MB-231 cells (**Figure 3B**), it resulted in the reduced expression of EMT genes upregulated by M protein, such as Twist (2.2-fold decrease), Zeb1 (7.4-fold decrease), HIF-1α (1.95-fold decrease), Snail (1.5-fold decrease) in MDA-MB-231 cells and Zeb1 (1.7-fold decrease) in MCF-7 cells (**Figure 3C**). In addition, treatment with a STAT3 inhibitor impaired the expression of mesenchymal markers, such as N-Cadherin (2.4-fold decrease), and Vimentin (2.9-fold decrease) in M protein-treated MDA-MB-231 cells (**Figure 3D**).

Next, we examined the role of the STAT3 pathway in the induced migratory ability of BCC by M protein. As a result, treatment with a STAT3 inhibitor significantly suppressed M protein-induced migration in both MDA-MB-231 and MCF-7 cells (MDA-MB-231: 2.9-fold decrease, MCF-7: 1.5-fold decrease, **Figure 3E**).

To examine the relationship between the NFκB and STAT3 pathways in M protein-treated MDA-MB-231 cells, we next examined the phosphorylation of STAT3 protein in M protein-treated MDA-MB-231 cells cultured in the presence of an NFκB inhibitor. As shown in **Figure 3F**, an NFκB inhibitor suppressed the phosphorylation of STAT3 in M protein-treated MDA-MB-231 cells (1.3-fold decrease, p<0.05), suggesting that the activation of the NFκB pathway might trigger the STAT3 pathway in M protein-treated MDA-MB-231 cells.

Taken together, these data suggested that, in MDA-MB-231 cells, M protein activated NFκB, consequently upregulating inflammatory cytokines and the STAT3 pathway, which are involved in the induction of EMT and migration. However, in MCF-7 cells, the activation of both the NFκB and STAT3 pathways was involved in the induction of the expression of EMT genes and migration.

### Promotion of Mobility, Proliferation and Stemness of MCF-7 Cells by SARS-CoV-2 M Protein-Treated MDA-MB-231 Cells

**Figure 4:**
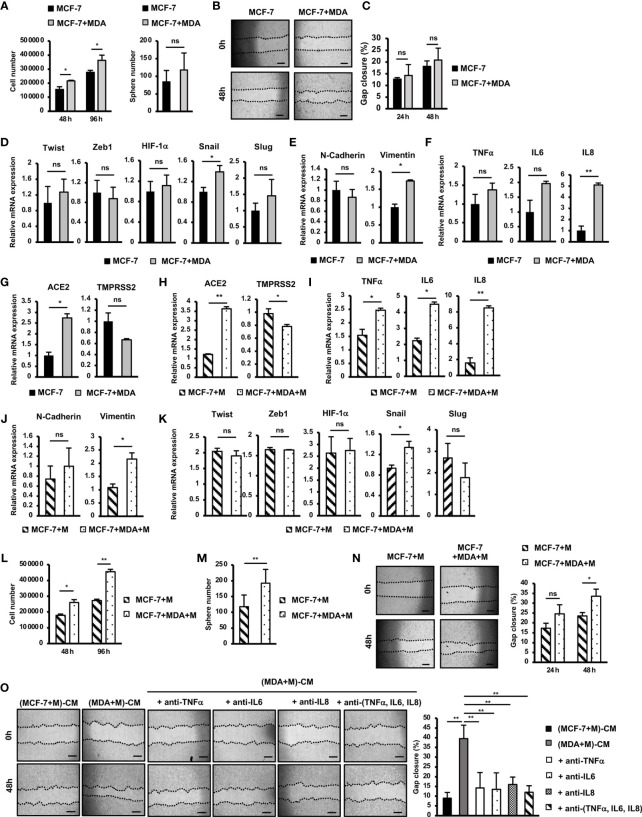
SARS-CoV-2 M protein-treated MDA-MB-231 cells promoted the mobility, proliferation and stemness of MCF-7 cells. **(A)** Proliferation of MCF-7 cells cocultured with MDA-MB-231 cells. **(B)** A mammosphere assay of MCF-7 cells cocultured with MDA-MB-231 cells. **(C)** Migration of MCF-7 cells cocultured with MDA-MB-231 cells. **(D)** The mRNA expression of genes related to EMT process in MCF-7 cells cocultured with MDA-MB-231 cells. **(E)** The mRNA expression of EMT markers in MCF-7 cells cocultured with MDA-MB-231 cells. **(F)** Gene expression of inflammatory cytokines in MCF-7 cells cocultured with MDA-MB-231 cells. **(G)** Gene expression of SARS-CoV-2 binding receptors in MCF-7 cells cocultured with MDA-MB-231 cells. **(H)** Gene expression of SARS-CoV-2 binding receptors in MCF-7 cells cocultured with M protein-treated MDA-MB-231 cells. **(I)** Gene expression of inflammatory cytokines in MCF-7 cells cocultured with M protein-treated MDA-MB-231 cells. **(J)** Gene expression of EMT markers in MCF-7 cells cocultured with M protein-treated MDA-MB-231 cells. **(K)** The mRNA expression of genes related to EMT process in MCF-7 cells cocultured with M protein-treated MDA-MB-231 cells. **(L)** Proliferation of MCF-7 cells cocultured with M protein-treated MDA-MB-231 cells. **(M)** Mammosphere assay of MCF-7 cells cocultured with M protein-treated MDA-MB-231 cells. **(N)** Migration of MCF-7 cells cocultured with M protein-treated MDA-MB-231 cells. **(O)** Migration of MCF-7 cells in conditioned medium (CM) from M protein-treated MCF7 cells or M protein-treated MDA-MB-231 cells in the presence of anti-TNFα antibody, anti-IL6 antibody and anti-IL8 antibody. The scale bars indicate 500 µm. Each value represents the mean ± SD of triplicate experiments. (ns, no significance; p > 0.05; *p ≤ 0.05; **p ≤ 0.01).

Tumors are a heterogenous mixture of different malignant and nonmalignant cells, in which non-aggressive cells can acquire new phenotypes through communication with aggressive cells, promoting malignancy (25). Therefore, to examine the effects of aggressive BCC on non-aggressive BCC, we next cocultured MCF-7 cells, a non-aggressive BCC line, with MDA-MB-231 cells, an aggressive BCC line, and characterized the altered phenotypes of MCF-7 cells. The results showed that coculturing with MDA-MB-231 cells significantly induced the proliferation of MCF-7 cells (1.3-fold increase after 72 h coculturing, **Figure 4A**) but showed no effects on the sphere formation or migration of MCF-7 cells (**Figures 4B, C**). We then examined the effects of MDA-MB-231 cells on the gene expression of MCF-7 cells. Coculturing with MDA-MB-231 cells significantly induced the expression of Snail (1.4-fold increase) but showed no effects on the expression of the other EMT genes (**Figure 4D**). In addition, coculturing with MDA-MB-231 cells upregulated the expression of Vimentin (1.7-fold increase, **Figure 4E**), a mesenchymal marker in EMT in MCF-7 cells. However, coculturing with MDA-MB-231 cells showed no effects on the expression of inflammatory cytokines, including TNFα, IL6 and IL8 in MCF-7 cells (**Figure 4F**). Interestingly, coculturing with MDA-MB-231 cells significantly upregulated the expression of ACE2 (2.74-fold increase, **Figure 4G**), a binding receptor of SARS-CoV-2.

Next, we examined the effects of M-protein on the ability of MDA-MB-231 cells to alter the phenotypes of MCF-7 cells. First, MCF-7 cells were cocultured with M protein-treated MDA-MB-231 cells, and then the gene expression was compared with that of MCF-7 cells treated with M protein directly. The results showed that coculturing with M protein-treated MDA-MB-231 cells induced the expression of ACE2, the SARS-CoV-2-binding receptor, in MCF-7 cells (3.6-fold increase, **Figure 4H**). Of note, coculturing with M protein-treated MDA-MB-231 cells upregulated the expression of inflammatory cytokines, which was not seen in MCF-7 cells treated with M protein directly (IL6: 2-fold increase, IL8: 5.3-fold increase, and TNFα:1.6-fold increased, **Figure 4I**). In addition, M protein-treated MDA-MB-231 cells significantly induced the expression of Vimentin (2.2-fold increase, **Figure 4J**), but showed no effects to upregulated other genes related to EMT in MCF-7 cells (**Figure 4K**).

We then examined the effects of M protein-treated MDA-MB-231 cells on the phenotypes of MCF-7 cells in comparison to MCF-7 cells treated with M protein directly. The results showed that M protein-treated MDA-MB-231 cells significantly induced the proliferation of MCF-7 cells (1.7-fold increase after 96 h of coculturing, **Figure 4L**). Notably, M protein-treated MDA-MB-231 cells showed the significant promotion of sphere formation (1.64-fold increase, **Figure 4M**) and migration (1.43-fold increase, **Figure 4N**) of MCF-7 cells, findings that were not observed in MCF-7 cells cocultured with MDA-MB-231 cells.

M protein-treated MDA-MB-231 cells showed the upregulation of inflammatory cytokines such as TNFα, IL6 and IL8 (**Figure 2A**), which were reported to be involved in the EMT and metastasis of BCC (26, 27). Therefore, we speculated the effects of these cytokines on the induced migration of MCF-7 cells by M protein-treated MDA-MB-231 cells. To check this hypothesis, the migration of MCF-7 cells in the conditioned medium-derived from M protein-treated MDA-MB-231 cells (CM) were compared with those in CM with the addition of neutralizing antibodies for TNFα, IL6, and IL8. As shown in **Figure 4O**, while CM significantly promoted the migration of MCF-7 cells, CM with the addition of neutralizing antibodies, either individually or together, showed no induced effects on the migration of MCF-7 cells. These data suggested that the upregulation of TNFα, IL6 and IL8 contributed to the induced paracrine effects of M protein-treated MDA-MB-231 cells on the migration of MCF-7 cells.

Taken together, these data suggested that, in addition to promoting the metastatic phenotypes of MDA-MB-231 cells, M protein also induces the paracrine effects of MDA-MB-231 cells on other non-aggressive BCC, thereby facilitating cancer progression. Specifically, M protein-treated MDA-MB-231 cells induced migration, proliferation and stemness, which might be involved in the upregulation of inflammatory cytokines and EMT genes, of MCF-7 cells.

## Discussion

Numerous studies have suggested that, in addition to having a high risk of SARS-CoV-2 infection, cancer patients might have an increased risk of accelerated cancer progression following infection (6–10). In addition, several case reports showed that breast cancer developed worsened outcomes after being infected by SARS-CoV-2, including new metastases and death due to tumor progression (8, 9). In the present study, our results demonstrated that SARS-CoV-2 M protein stimulated the migration, invasion and expression of EMT genes in both MDA-MB-231 cells, a TNBC cell line, and MCF-7 cells, a hormone-dependent BCC line (28, 29). These results were in line with those of a previous study in which sera from COVID-19 patients induced EMT and Vimentin, Zeb1 and Snail expression in lung, breast and colon cancer cells *in vitro* (10).

Previous study suggested that breast tumor tissues from TNBC patients showed the expression of ACE2, a receptor of SARS-CoV-2 (30). In the present study, we found that while MCF-7 cells showed the low expression of ACE2 and TMPRSS2 on the cell membrane surfaces, MDA-MD-231 cells exhibited the high expression of these receptors (**Supplementary Figure 1**). Among subtypes of breast cancer, TNBC is an aggressive type with a poor prognosis and low efficacy of targeted treatment (31). This raises concerns that cancer progression might be exacerbated when TNBC patients are infected with SARS-CoV-2. Of note, our finding suggested that MDA-MB-231 cells, but not MCF-7 cells, showed the induced aggressive phenotypes, including proliferation, stemness and *in vivo* metastasis by M protein. Therefore, it is necessary to perform further studies with a long-term follow-up of TNBC patients after SARS-CoV-2 infection.

In BCC, the activation of NFκB, a proinflammatory transcription factor, drives the inflammatory responses, proliferation, migration and invasion, leading to cancer development and progression. NFκB is also involved in the expansion of breast cancer stem cells, which are intimately associated with cancer relapse and metastasis. In clinical studies, the enhanced activation of NFκB is associated with the breast tumor size, malignant progression, aggressive behavior and metastases in breast cancer (23). A previous study reported that M protein of coronaviruses triggered the NFκB signaling pathway in MDA-MB-231 cells (32). Consistently, our study showed that M protein of SARS-CoV-2 activated the NFκB pathway, which is responsible for the upregulation of EMT and tumor progression-related genes, such as Zeb1/2, Snail, Twist and HIF-1α, in MDA-MB-231 cells (33). In addition, the activation of the NFκB pathway in M protein-treated MDA-MB-231 cells also induced the expression of inflammatory cytokines, including IL6, IL8, and TNFα, which are involved in tumor initiation and homing and metastasis of BCC (34–36) and might contribute to the amplification of the cytokine storm.

Numerous studies reported that STAT3, a signaling pathway associated with migration, invasion and cell plasticity, is associated with the NFκB pathway and stem-like phenotype of BCC (24, 37, 38). In addition, STAT3 was reported to be activated by inflammatory cytokines, such as IL6, IL8 and TNFα, which enhance breast cancer proliferation, invasion and metastasis through the upregulation of Twist, Snail, Slug, Vimentin and HIF-1α (24). In the present study, our findings suggested crosstalk between the NFκB and Jak/STAT3 signaling pathways through the autocrine expression of IL6, IL8 and TNFα in MDA-MB-231 cells induced by SARS-CoV-2 M protein. Therefore, the NFκB and Jak/STAT3 signaling pathways might be promising targets of treatment for TNBC patients who develop COVID-19 infection.

**Figure 5:**
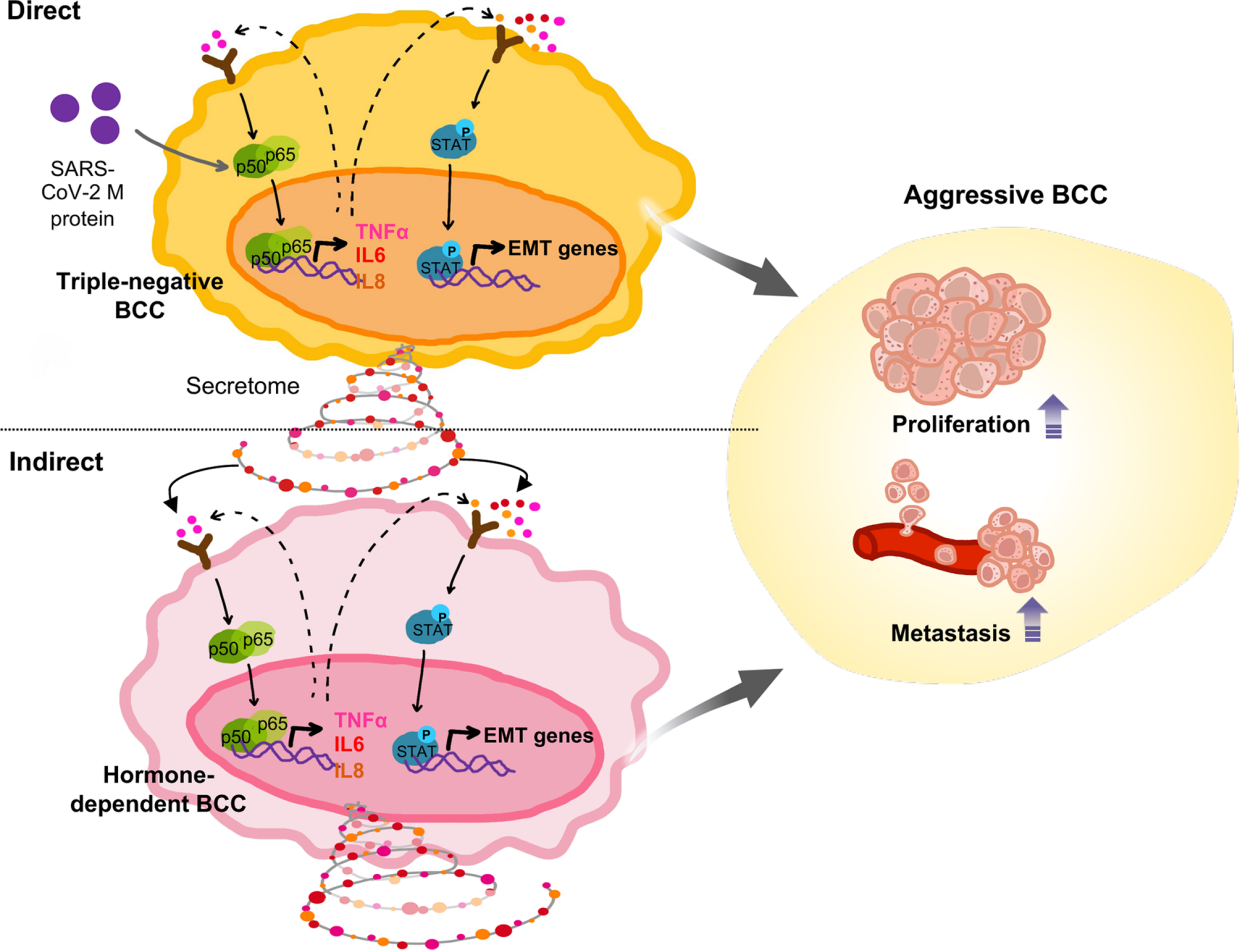
Proposed model: In TNBC cells, SARS-CoV-2 M protein activated the NFκB and STAT3 signaling pathways, which are involved in mobility, proliferation and stemness, thereby facilitating *in vivo* metastasis. In addition, SARS-CoV-2 M protein-treated TNBC cells caused malignant transformation of non-aggressive BCC.

Tumors are a heterogenous mixture of cancer cells, in which non-aggressive cells can acquire new phenotypes such as malignancy through communication with aggressive cells (25). By coculturing of MCF-7, as a non-aggressive BCC, with MDA-MB-231, as an aggressive BCC, we found that MDA-MB-231 cells induced the proliferation, migration, and the expression of Vimentin, a mesenchymal marker, of MCF-7 cells. Our data are in line with those of previous studies which suggested that TNBC cells induce other subtypes of BCC to transform to an aggressive phenotype (39, 40). Of note, our findings suggested that M protein induction amplified the ability of MDA-MB-231 cells to induce the transition to an aggressive phenotype of MCF-7 cells, including the migration, proliferation, stemness and inflammatory cytokine expression, which were not happened in MCF-7 cells directly treated by M protein (**Figure 5**). Interestingly, our findings showed that the coculture of MCF-7 cells with M protein-treated MDA-MB-231 cells significantly induced the expression of ACE2 in MCF-7 cells. As ACE2 also serves as a biomarker of EMT and metastasis (15, 41), these data hinted that the upregulation of ACE2 by M protein-induced aggresstive BCC might facilitate the infection of SARS-CoV-2 and metastasis in non-aggressive BCC; suggesting that, in the heterogenous mixture of cells inside tumors, SARS-CoV-2-infected aggressive BCC may affect non-aggressive BCC through secretome and cytokine storm and promote a poor general outcome of tumor progression. Our present data were based on established cell lines instead of patient samples; therefore, it is worth to investigate the effects of SARS-CoV-2 infection on the interaction between aggressive BCC and non-aggressive BCC derived from breast cancer patients.

Numerous reports showed the existence of SARS-CoV-2 viral proteins in sera of COVID-19 patients (42–45), suggesting that in addition to the direct infection of SARS-CoV-2 into cells, free viral proteins of SARS-CoV-2 in sera might also affect the surrounding cells. However, although several studies reported the effects of SARS-CoV-2 proteins on numerous types of cells (46–48), how SARS-CoV-2 protein, such as M protein, gets internalized into the cell is still obscured. Therefore, it is noteworthy for a further study to examine whether M protein binds to a specific receptor in the membrane surface of breast cancer cells or is non-selectively internalized into cells through macropinocytosis (49).

## Conclusion

In summary, the present study demonstrated the effects of SARS-CoV-2 M protein on the malignant phenotypes of TNBC MDA-MB-231 cells, including the invasion, proliferation, stemness and *in vivo* metastasis of TNBC MDA-MB-231 cells, which might be involved in the upregulation of EMT genes regulated by the NFκB and Jak/STAT3 signaling pathways. Of note, M protein promoted the ability of MDA-MB-231 cells to induce malignant phenotypes in nonaggressive BCC lines, such as the hormone-dependent line MCF-7. Therefore, our findings suggested an increased risk of poor outcomes in breast cancer patients following SARS-CoV-2 infection, which should be noted while caring for cancer patients with COVID-19.

## Data Availability Statement

The raw data supporting the conclusions of this article will be made available by the authors, without undue reservation.

## Ethics Statement

The animal study was reviewed and approved by The Animal Care Committee of the University of Tsukuba.

## Author Contributions

H-NN contributed to the study concept, conducted the experiments and data analysis, and wrote the original draft of manuscript. MK contributed to the experiments, interpretation and data analysis. MF and C-KV contributed to the study concept, writing and editing of the manuscript. TY contributed to the *in vivo* experiments and technical support. OO raised the study concept and design, editing of the manuscript and final approval. All authors read and approved the final manuscript.

## Conflict of Interest

The authors declare that the research was conducted in the absence of any commercial or financial relationships that could be construed as a potential conflict of interest.

## Publisher’s Note

All claims expressed in this article are solely those of the authors and do not necessarily represent those of their affiliated organizations, or those of the publisher, the editors and the reviewers. Any product that may be evaluated in this article, or claim that may be made by its manufacturer, is not guaranteed or endorsed by the publisher.

## Supplementary Material

Supplementary Figure 1 MDA-MB-231 cells showed higher expression of SARS-CoV2 binding receptors, ACE2 and TMPRSS2, in comparison to MCF-7 cells. BCC were stained with primary antibodies, including rabbit Anti-ACE2 antibody (GTX101395, Genetex, Irvine, CA, USA) or rabbit Anti-TMPRSS2 antibody (GTX81494, Genetex) following by secondary antibody Goat Anti-rabbit Igg DyLight488 (GTX213110-04, Genetex). The expression of ACE2 and TMPRSS2 was examined by fluorescence activated cell sorting (FACS) analysis.

Supplementary Figure 2 Full-length blots of Western blot figures. (A) Protein expression of p65 and p50 in MDA-MB-231 and MCF7 cells, which shown in Figure 2B. (B) The phosphorylation of STAT3 protein in MDA-MB-231 and MCF7 cells, which shown in Figure 3A. (C) The phosphorylation of STAT3 protein in MDA-MB-231 and MCF7 cells in the present of NFκB inhibitor, which shown in Figure 3F.
