# Supplementary figures and images for: SARS-CoV-2 M Protein Facilitates Malignant Transformation of Breast Cancer Cells
Source: Front Oncol. 2022 Jun 7;12:923467. doi: 10.3389/fonc.2022.923467 (PMC9209714; doi:10.3389/fonc.2022.923467)

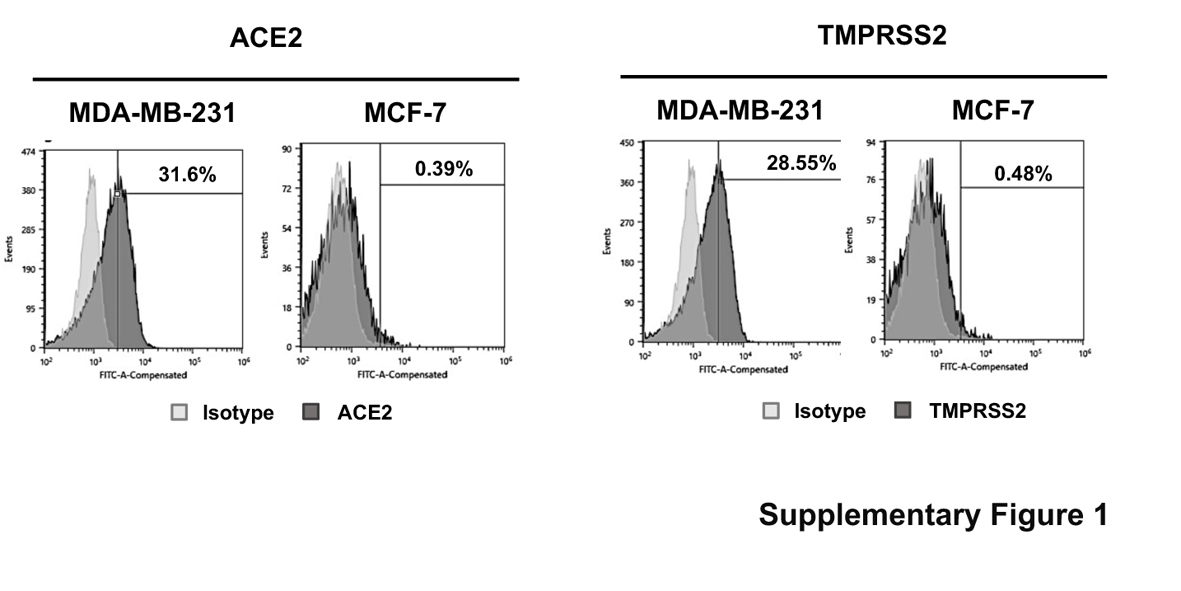

Supplement: Supplementary Figure 1 — MDA-MB-231 cells showed higher expression of SARS-CoV2 binding receptors, ACE2 and TMPRSS2, in comparison to MCF-7 cells. BCC were stained with primary antibodies, including rabbit Anti-ACE2 antibody (GTX101395, Genetex, Irvine, CA, USA) or rabbit Anti-TMPRSS2 antibody (GTX81494, Genetex) following by secondary antibody Goat Anti-rabbit Igg DyLight488 (GTX213110-04, Genetex). The expression of ACE2 and TMPRSS2 was examined by fluorescence activated cell sorting (FACS) analysis. [file Image_1.tif]

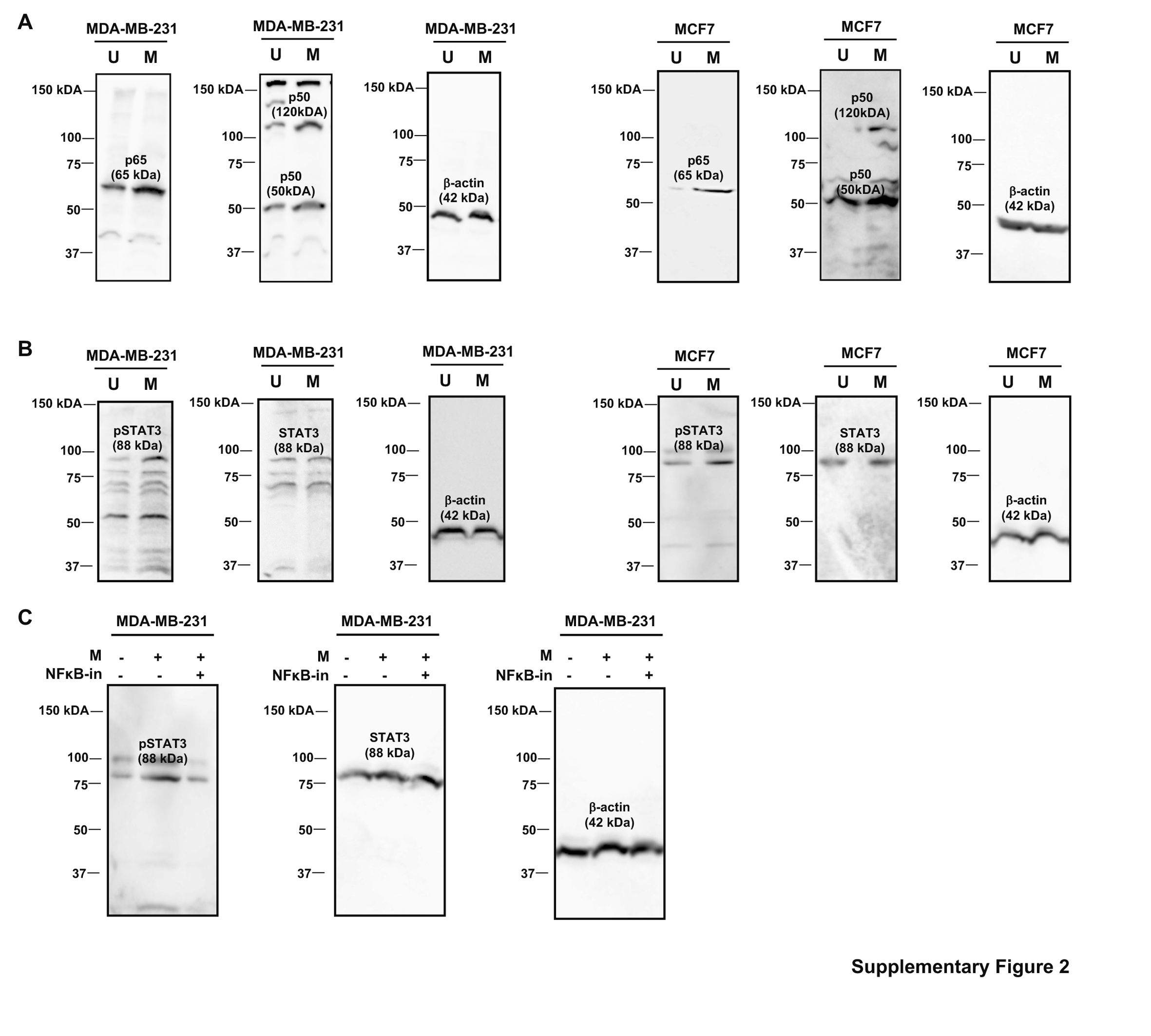

Supplement: Supplementary Figure 2 — Full-length blots of Western blot figures. (A) Protein expression of p65 and p50 in MDA-MB-231 and MCF7 cells, which shown in Figure 2B. (B) The phosphorylation of STAT3 protein in MDA-MB-231 and MCF7 cells, which shown in Figure 3A. (C) The phosphorylation of STAT3 protein in MDA-MB-231 and MCF7 cells in the present of NFκB inhibitor, which shown in Figure 3F. [file Image_2.tif]
